# Traditional use of benznidazole with weekly clinical follow-up indicate to be an important approach for the etiologic treatment of Chagas disease

**DOI:** 10.1590/0074-02760210034chgsa

**Published:** 2022-07-08

**Authors:** Gilberto Marcelo Sperandio da Silva

**Affiliations:** 1 Fundação Oswaldo Cruz-Fiocruz

Despite more than 100 years of the description of Chagas disease (CD), it still affects about 6 to 7 million people worldwide, most of them, about 6 million in Latin America.^1^ ^,^ ^2^

CD can be associated with situations of poverty, when the kissing bug invades the house and feeds on the human host, infecting it with the flagellate protozoan *Trypanosoma cruzi*, the agent of CD. The CD estimated prevalence in Brazil for 2020 is about 1.3 to 3.2 million individuals.^3^ CD has two phases: acute and chronic. The acute phase of the disease is generally subclinical and lasts approximately 8 to 12 weeks. After the acute phase, patients move on to the chronic phase characterised by low and intermittent parasitaemia and approximately 30-40% will progress to the cardiac form and 10% to the digestive.

In general, benznidazole (BZN) is the most used drug for etiologic treatment in Brazil, indicated for all age groups when the patient is in the acute phase and, in the chronic phase, for individuals under 50 years of age without advanced heart disease. Nifurtimox (NFx) would be an alternative for patients who are intolerant to BZN. The standard dosage of BZN for children is 5 to 10 mg/kg/day, divided into two daily doses, and for adults, it is 5 mg/kg/day. The maximum daily dose is 300 mg and the minimum treatment time is 60 days. This treatment time can be extended up to 80 days in patients over 60 kg.^1^

Recently, Torrico et al.^4^ compared several treatment regimens involving BZN, among them 300mg/day for two, four, and eight weeks. There were no differences in terms of efficacy for shortened treatments with BZN. However, there were two serious adverse events (leukopenia and neutropenia) in patients treated for eight weeks. In the 2-week treatment regimen, no serious adverse event was observed,^4^ perhaps due to the limited sample size (30 individuals per group). Also, within this context, we observed that dermatological disorders represent the most common adverse event of BZN and their onset occurs around the first 10 days of treatment. Therefore, shortening the treatment may not solve this limitation of BZN. On this topic, we suggest that BZN needs to be followed up intensively, preferably weekly, as 0.1% of skin reactions can be severe.^5^ Additionally, a study demonstrated that BZN has a clinical benefit in patients in the indeterminate chronic phase and younger than 50 years of age, reducing the risk for progression to the cardiac form of the disease, and cardiovascular events. This study suggests that the use of BZN be implemented in clinical practice for the etiological treatment of patients with the indeterminate form of CD for individuals up to 50 years of age.^6^ Additionally, another observational study that followed patients treated for up to 30 years suggests that the regimens proposed in the PCDT/2018 (300 mg for 60 days) are more effective in decreasing indirect immunofluorescence serological titers when compared with shorter-term regimens.^7^ New approaches and associations of BZN with other drugs become necessary for future clinical trials.

Comments on the article: Mendes FSNS, Perez-Molina JA, Angheben A, Meymandi SK, Sosa-Estani S, Molina I. Critical analysis of Chagas disease treatment in different countries. Mem Inst Oswaldo Cruz. 2022; 117: e210034.
